# Chemical Stability between NiCr_2_O_4_ Material and Molten Calcium-Magnesium-Alumino-Silicate (CMAS) at High Temperature

**DOI:** 10.3390/ma10121397

**Published:** 2017-12-06

**Authors:** Zhuang Ma, Xing Li, Ling Liu, Yanbo Liu

**Affiliations:** 1 School of Materials Science and Engineering, Beijing Institute of Technology, Beijing 100081, China; hstrong929@bit.edu.cn (Z.M.); lixingldy@163.com (X.L.); lyb197896@bit.edu.cn (Y.L.); 2 National Key Laboratory of Science and Technology on Materials under Shock and Impact, Beijing 100081, China

**Keywords:** CMAS attack, impermeable protective coating, chemical stability

## Abstract

NiCr_2_O_4_ as a potential protection for thermal barrier coatings (TBCs) against the attack of molten calcium-magnesium-alumino-silicate (CMAS) was studied by a CMAS-contacting experiment. Atmospheric plasma sprayed coatings and sintered bulk materials were fabricated, covered with CMAS deposits, and exposed to 1200 °C for 24 h. Nano-sized CMAS-NiCr_2_O_4_ mixed powder was manufactured by ball milling and then conducted heat treatment under the same condition. The results show that no reacting product was found at the border between molten CMAS and NiCr_2_O_4_ and no element transportation occurred. It can be inferred that NiCr_2_O_4_ has outstanding chemical stability with the molten CMAS.

## 1. Introduction

The use of ceramic thermal barrier coatings (TBCs) on hot-section metallic components in gas-turbine engines used to propel aircraft enabled it to operate at higher temperatures [1,2,3], which in turn has generated several new issues. One of them is the degradation and spallation of the TBCs caused by melting calcium-magnesium-alumino-silicate (CMAS) deposits, which originates from sand, dust, fly ash, and volcano ash on the hot TBC surface [4,5]. Typical 7 wt % Y_2_O_3_-stabilized ZrO_2_ (7YSZ), which is widely used as a TBC material, is highly susceptible to CMAS attack at high temperatures. The molten CMAS can penetrate the 7YSZ coating through its pores/cracks, and the 7YSZ grains can be dissolved in molten CMAS. The relatively low solubility of Zr^4+^ in molten CMAS compared with Y^3+^ leads to the reprecipitation of Y-depleted ZrO_2_ grains [6]. During the cooling stage of the engine, the destabilized ZrO_2_ grains transform from tetragonal phase to monoclinic phase and this period is combined with significant volume expansion, which could lead to the delamination and spalling of the TBC [7]. This issue has attracted a lot of attention in recent years, because the attack caused by molten CMAS decreases the service life of the engine by about a half [8].

For the protection of TBCs from molten CMAS attack, many methods have been applied. According to the patents presented by Hasz et al., the protective coating against CMAS attack can be assorted into three types, i.e., impermeable, sacrificial, and nonwetting types [9,10,11]. Nowadays, the research emphasis in this field has been put on sacrificial and impermeable protective coatings. Rai et al. [12] investigated dense Pt film as a protective layer that renders TBCs impermeable to CMAS attack. L. Wang et al. compared Pt film and Gd_2_Zr_2_O_7_ (GZO) as typical sacrificial coatings in a molten CMAS penetration test [13], and the result showed that Pt film exhibited much better anti-CMAS ability than GZO coating. However, the mismatch between Pt with ceramic TBCs restricts its efficacy in common use.

It is obvious that finding some ceramic impermeable protective coatings is vital for the protection of TBCs from CMAS attack. In the analysis of reacting product of bond coating material (NiCrAlY) and substrate material for TBC (nickel-based super alloy Inconel 738 material) reacting with CMAS, C.S. Ramachandran et al. [14] found that NiCr_2_O_4_ formed in the reactive layer as a reactive product. On the other hand, optical basicity (OB) [15,16], which was first reported by Duffy et al. [17], based on Lewis acid-base theory, can be used to determine the chemical stability between two materials. The difference between the OBs (Δ∧) of CMAS and coating material can be regarded as the reacting ability of molten CMAS infiltration reaction, where a higher Δ∧ infers more reactivity. It can be inferred that NiCr_2_O_4_ (Δ∧: 0.75) and CMAS (Δ∧: 0.65) [18] have favorable chemical stability because their OBs are relative close. In addition, the use of NiCr_2_O_4_ on TBCs will not increase the thermal conductivity too much because the thermal conductivity of NiCr_2_O_4_ is 3.3 Wm^−1^K^−1^.

In this work, in order to obtain a potential impermeable top layer material for the protection of TBCs, we conducted high-temperature interactions of molten CMAS contact with three different types of NiCr_2_O_4_ to investigate their chemical stability: coatings, bulk samples, and original powders. These three types have different research targets: coating samples are used to test their interaction with the morphology adjusting to the reality; bulk samples are used to achieve the pure phase of NiCr_2_O_4_ to omit the influence of impurities that are easily contained in coating samples; and original powders are made to obtain a sample with NiCr_2_O_4_ and CMAS contacted extensively at the nano-scale.

## 2. Experiment

A simulated CMAS glass frit of composition 39.2 CaO-5.2 MgO-4.1 Al_2_O_3_-51.5 SiO_2_ (mol %) was prepared for the molten CMAS penetration experiment. This CMAS composition is similar to typical sand deposits that are found in engines. The CMAS glass was synthesized by mixing single oxides according to a method recorded elsewhere [6].

The original NiCr_2_O_4_ powder was synthesized by the solid phase method under 1200 °C. NiCr_2_O_4_ powders with a spherical shape and proper flowability were fabricated by dried spraying technology. A NiCr_2_O_4_ coating with porosity of 4.6% was sprayed on a series of stainless steel substrates (22 mm long, 8 mm wide, 2.8 mm thick), grit-blasted by atmospheric plasma spraying (APS) using a SG100 plasma gun (Praxair, Danbury, CT, USA). The parameters used in the plasma spray process are listed in Table 1. For of high temperature heat treatment with CMAS, the substrates coated with NiCr_2_O_4_ were bent by pliers to obtain free-standing coatings with a thickness of 0.4 mm. A series of NiCr_2_O_4_ bulk samples with 84.6% density were formed by solid sintering (1600 °C, 10 h).

The simulated CMAS glass frit was mixed in alcohol (AR500, Weizhicheng Chemical Ltd., Nanjing, China) and then distributed on the upper surface of the free-standing NiCr_2_O_4_ coating and NiCr_2_O_4_ bulk sample with a concentration of 35 mg/cm^2^. After the alcohol evaporated, both the bulk sample and free-standing NiCr_2_O_4_ coating with the well-distributed CMAS glass frit cover were heat-treated in an electric furnace at 1200 °C for 24 h. A sample of 50%CMAS-50%NiCr_2_O_4_ original powder was made by ball milling and then heat-treated under 1200 °C for 24 h. Both NiCr_2_O_4_ and CMAS powders were ball milled with 400 rpm for 6 h to achieve nano-size particles and then mixed well to form CMAS-NiCr_2_O_4_ with a relatively large interface between the two materials.

The phase analysis of the NiCr_2_O_4_ bulk sample and coating was conducted by X-ray diffraction (XRD, RIGAKU D/Max-rB, Rigaku International Corp., Tokyo, Japan) with Cu*Kα* radiation at a scan rate of 4°/min. The cross-section samples were polished and analyzed by using scanning electron microscopy (SEM, S-4800, Hitachi Ltd., Ibaraki, Japan) equipped with an energy dispersive spectrometer (EDS, D-8 advance, Bruker, Karlsruhe, Germany) for elemental analysis, and both operated at 10 kV accelerating voltage. Raman spectroscopy (HR800, JobinYvon Horiba, Oberursel, Germany) with a 532-nm exciting wavelength was also used in the analysis of the distribution of materials in the bulk samples CMAS-contacting experiment. The beam power was 14 mW and the spectral resolution was 3.5 cm^−1^. The microstructure of 50%CMAS-50%NiCr_2_O_4_ original powder was analyzed with a transmission electron microscope (TEM, Tecnai F20, Waltham, MA, USA) with an energy dispersive spectrometer (EDS) and selected area electron diffraction (SAED, Tecnai F20, Waltham, MA, USA) equipment. All were operated at 200 kV accelerating voltage.

## 3. Results and Discussion

**Figure 1:**
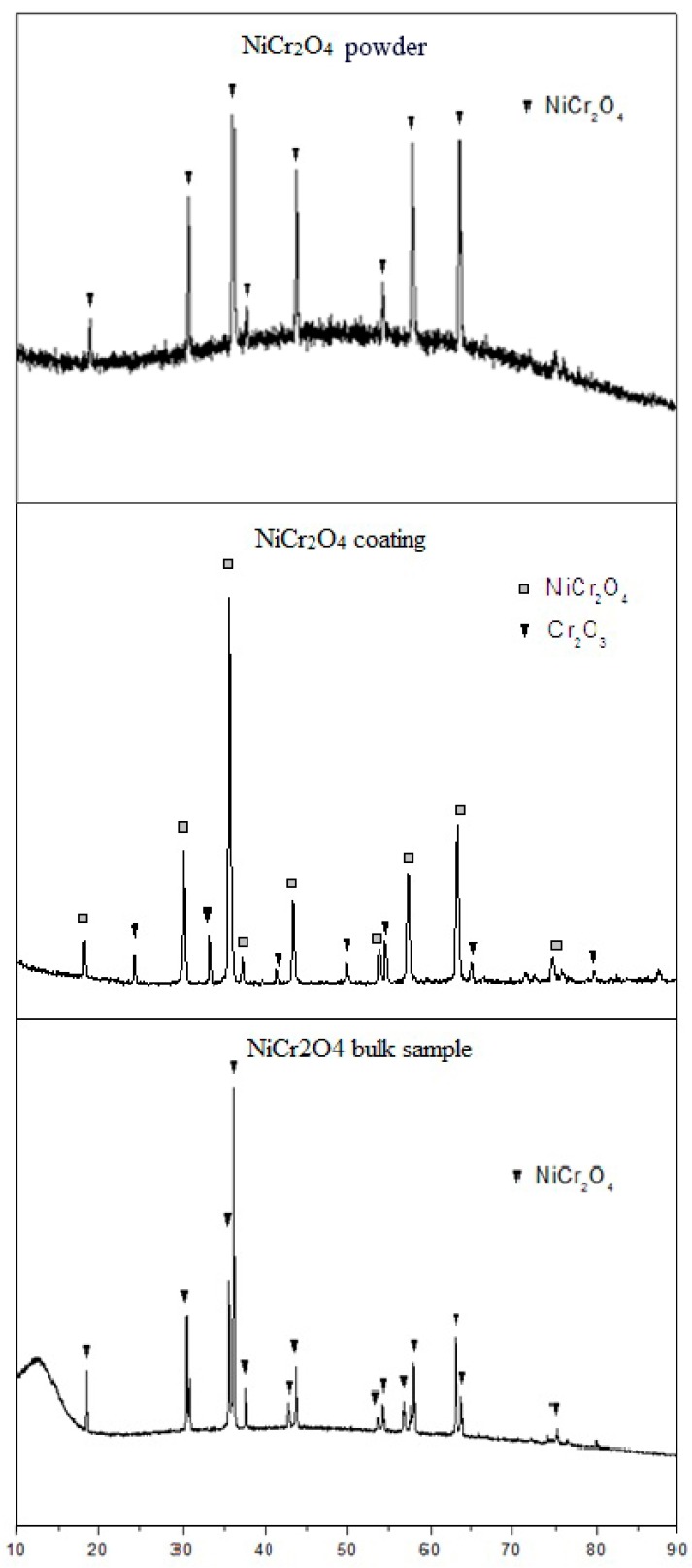
XRD patterns of NiCr_2_O_4_ original powder, coating, and bulk sample.

Figure 1 shows the XRD patterns of NiCr_2_O_4_ original powder, coating, and bulk sample. The XRD pattern of NiCr_2_O_4_ original powder (after sintering at 1200 °C) coincides with the standard spectrum of NiCr_2_O_4_ with a spinal structure (23-1271) in space group of Fd-3m. The XRD pattern of NiCr_2_O_4_ coating exhibits peaks corresponding to the mainly spinal structure NiCr_2_O_4_ as well as peaks corresponding to a small amount of Cr_2_O_3_ (about 18.4%, according to intensity of the peaks). It is suggested that the decomposition of the NiCr_2_O_4_ occurred during the APS procedure. The XRD patterns show that the NiCr_2_O_4_ bulk samples consist of pure NiCr_2_O_4_ phase in the space group of I41/amd. It can be inferred that sintering at 1600 °C changes the structure of NiCr_2_O_4_.

**Figure 2:**
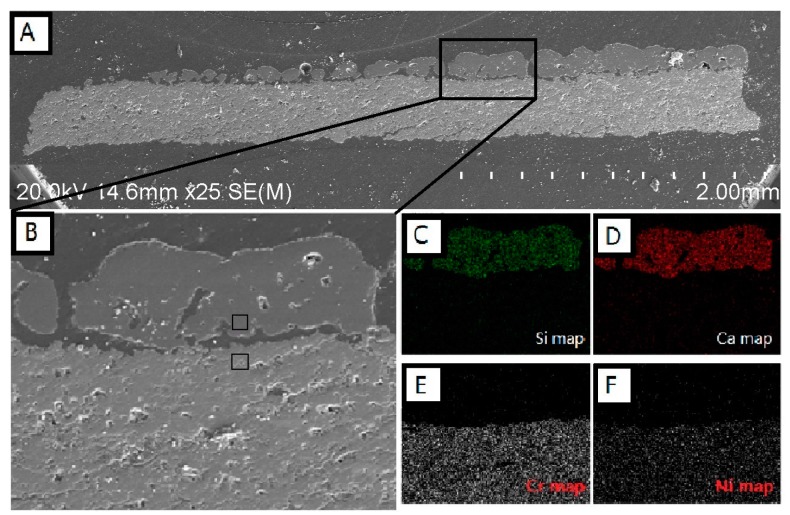
(**A**) Cross-sectional SEM micrographs of APS NiCr_2_O_4_ coating that interacted with 35 mg/cm^2^ CMAS at 1200 °C for 24 h; (**B**) Higher magnification image of CMAS-NiCr_2_O_4_ boundary and its (**C**) Si; (**D**) Ca; (**E**) Cr and (**F**) Ni maps.

Figure 2A shows cross-sectional SEM micrographs of APS NiCr_2_O_4_ coating interacted with CMAS. It shows that the CMAS deposit on the surface of the NiCr_2_O_4_ coating is fully melted under the heat treatment of 1200 °C for 24 h. Figure 2B shows that the microstructure, including the pores and lamellar structure of the NiCr_2_O_4_ coating, remained after CMAS interaction. There was no penetration of molten CMAS found at the border. Molten CMAS seems to be non-wetting with the NiCr_2_O_4_ coating, because the wetting angles of residue CMAS are obtuse angles and many spherical CMAS residues could be found on the surface of the NiCr_2_O_4_ coating. Figure 2B depicts an SEM image of the cross-section of the NiCr_2_O_4_ coating interacted with 35 mg/cm^2^ CMAS. Figure 2C−F are the EDS elemental maps of Si, Ca, Cr, and Ni in Figure 2B. It can be seen in the elemental maps that the interface between CMAS and NiCr_2_O_4_ is clear, and no elemental transportation gradient was observed from the border to each side. EDS cation compositions (at %) of the upper square (residue CMAS) and lower square (edge of the NiCr_2_O_4_ coating) in Figure 2B are listed in Table 2. The atomic ratio of Ni/Cr in the NiCr_2_O_4_ coating was 0.36 and the theoretical atomic ratio was 0.5. The atomic ratio of Ca/Si in residue CMAS on the surface was also similar to the composition of the simulated CMAS glass frit. The element distributions of both NiCr_2_O_4_ and CMAS after the heat treatment are similar to their original states. 

**Figure 3:**
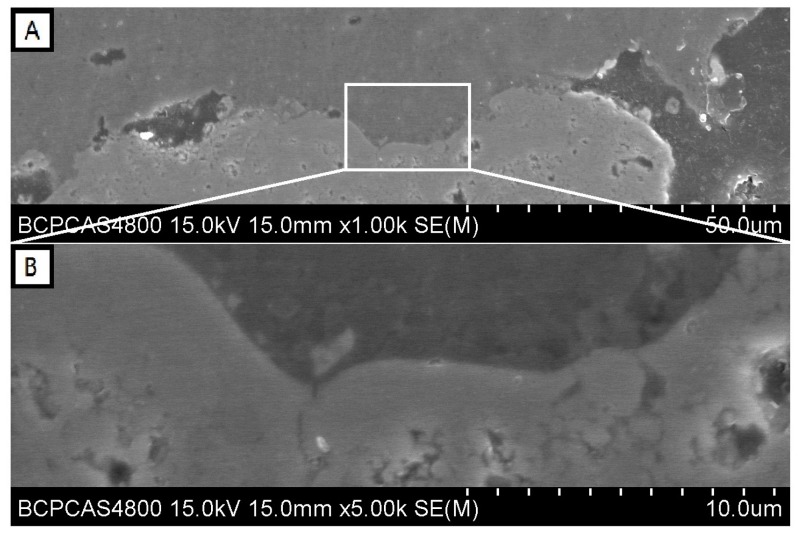
(**A**) Contact area of the CMAS-NiCr_2_O_4_ boundary and (**B**) its high magnification image.

Figure 3A is a high magnification SEM image of the border of interaction between residue CMAS and NiCr_2_O_4_ coating. The upper area is CMAS and the lower area shows the structure of the NiCr_2_O_4_ coating. At the contacted part of the border shown in Figure 3A,B, a split line with a width of less than 1 μm can be seen. The micro pores in the NiCr_2_O_4_ coating with a size of 3–5 μm are not filled and there is no any reaction product at the border. Some NiCr_2_O_4_ particles could be found at the edge of the residue CMAS, but no dissolving phenomenon of these particles was detected.

**Figure 4:**
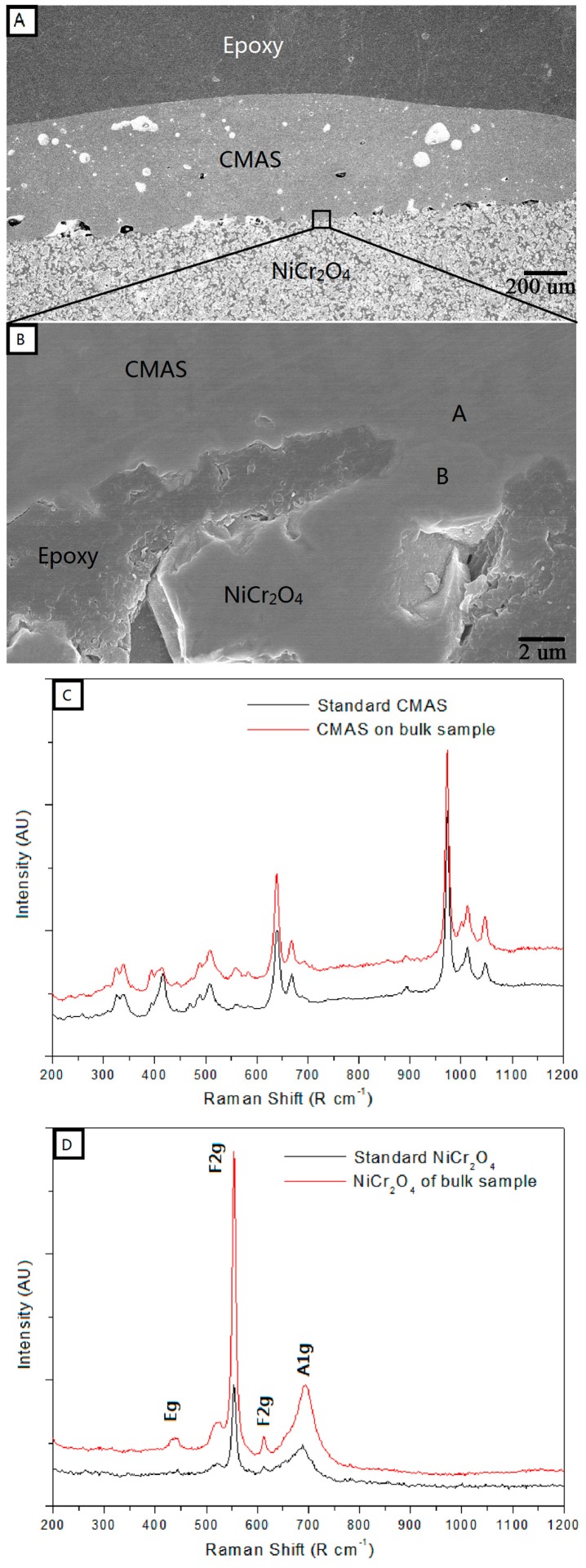
(**A**) Cross-sectional SEM micrographs of the NiCr_2_O_4_ bulk sample that interacted with 35 mg/cm^2^ CMAS at 1200 °C for 24 h and (**B**) a high magnification image of the CMAS-NiCr_2_O_4_ boundary; (**C**) Raman spectra at the area marked A in (**B**) compared with standard CMAS and (**D**) at the area marked B in (**B**) compared with standard NiCr_2_O_4_.

Figure 4A,B are cross-sectional SEM micrographs (low and high magnification) of NiCr_2_O_4_ bulk sample that were heat-treated with 35 mg/cm^2^ CMAS coating. The NiCr_2_O_4_ bulk sample has a high porosity and the pores were filled with epoxy during the SEM sample preparation. The CMAS did not infiltrate the bulk sample, but just melted and spread on the surface. The distance is less than 2 μm between letters A and B indicated in the figure, areas for which Raman spectroscopy was conducted. Figure 4C,D show the Raman spectra of CMAS and NiCr_2_O_4_ bulk samples that were marked A and B in Figure 4B. The compared standard materials of CMAS and NiCr_2_O_4_ are original powders that were heat-treated under the same conditions of 1200 °C for 24 h. The results show that the Raman spectra of the CMAS in the contact sample (area A) have similar peaks compared to the standard CMAS, and the Raman spectra of NiCr_2_O_4_ bulk samples (area B) are similar to the standard NiCr_2_O_4_. However, the peaks of the Raman spectra for CMAS and NiCr_2_O_4_ are completely different.

It was found that both the bulk sample and free-standing coating of NiCr_2_O_4_ were non-wetting with CMAS under 1200 °C. Because the morphologies of the coating samples and bulk samples are quite different and the results of CMAS contacting experiments for both the coating sample and bulk sample are similar, it can be inferred that morphology has less influence on the CMAS barrier ability for NiCr_2_O_4_ material when the CMAS has a non-wetting surface.

The XRD result of NiCr_2_O_4_ coating shows that Cr_2_O_3_ was found in the coatings, mainly because of the melting and recrystallization during the APS procedure. It is obvious that the residue Cr_2_O_3_ does not affect the arresting ability of the coating, probably because the optical basicity of Cr_2_O_3_ (Δ∧: 0.70) is similar to that of NiCr_2_O_4_, so we can assume that it has a reacting ability with CMAS similar to NiCr_2_O_4_. 

For remove of the influence of Cr_2_O_3_ impurities, NiCr_2_O_4_ bulk samples were fabricated by solid sintering and used to react with CMAS coating under the same conditions. The Raman spectra of CMAS and NiCr_2_O_4_ bulk samples were compared with a standard material, as shown in Figure 4, which gives us a new way to identify the phase structure and materials found at the micro-scale, such as at the border between CMAS and NiCr_2_O_4_ bulk samples. The NiCr_2_O_4_ bulk sample exhibited a tetragonal structure belonging to the space group I41/amd. The factor group analysis predicted the following modes in NiCr_2_O_4_:

2A_1g_(R) + B_2g_(R) + 3B_1g_(R) + 4E_g_(R) + A_2g_ + 2B_1u_ + 2A_1u_ + 4B_2u_ + 6E_2u_(IR) + 4A_2u_(IR) + B_1u_ [19].

There are 10 Raman active modes (2A_1g_ + B_2g_ + 3B_1g_ + 4E_g_) for the tetragonal structure of NiCr_2_O_4_, and some were detected in both area B in Figure 3B and in the the standard NiCr_2_O_4_ material. In contrast, the Raman spectra of area A in Figure 4B and of the pure CMAS conducted under the same heat treatment without any reaction showed high consistency, as the crystallization of CMAS occurred without any Raman active modes of NiCr_2_O_4_ in it. These results indicate that NiCr_2_O_4_ has no reaction with the CMAS bulk sample under 1200 °C, and the material transportation at border of the two materials is negligible (less than 2 μm).

**Figure 5:**
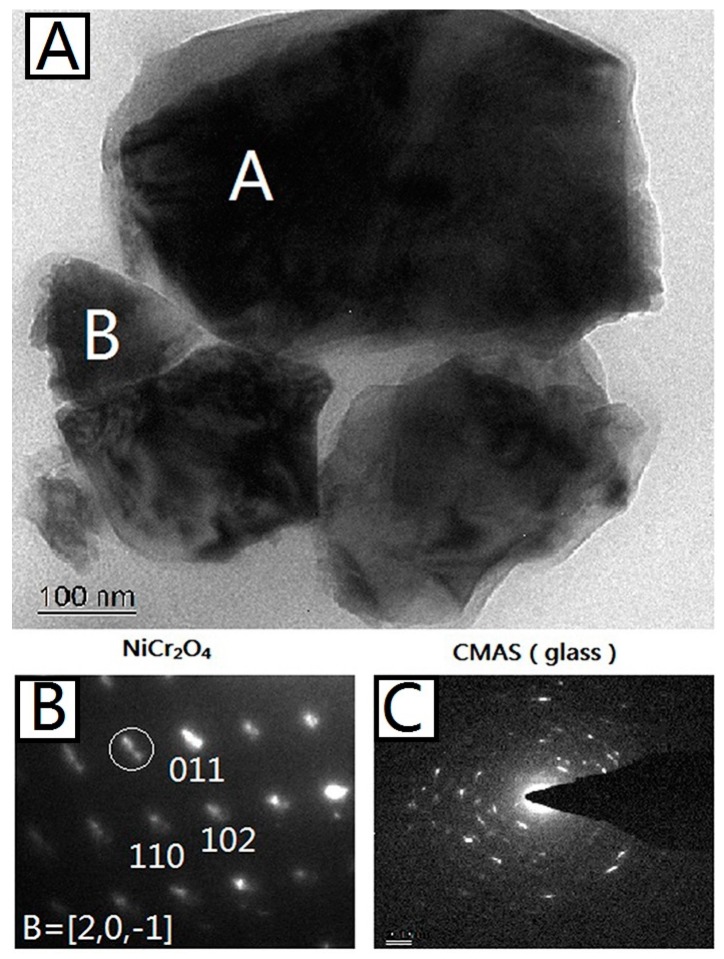
(**A**) Bright-field TEM micrograph of 50%CMAS-50%NiCr_2_O_4_ powder interacted under 1200 °C for 24 h. Phase-identified NiCr_2_O_4_ and CMAS (glass) grains are marked; (**B**) Indexed SAEDP of NiCr_2_O_4_ marked with A in (**A**); (**C**) Indexed SAEDP of CMAS marked with B in (**A**).

Figure 5A shows a bright-field TEM image of 50%CMAS-50%NiCr_2_O_4_ powder interacted under 1200 °C for 24 h. The grain marked A was identified as NiCr_2_O_4_ using SAEDP with a best match with PDF (Powder Diffraction File) No. 23-0423 for ideal composition in the space group of I41/amd. The grain marked B was identified as a glass structure of CMAS as its SAEDP showed a typical circle pattern for amorphous glass that contains several crystals separated out from CMAS. The elemental composition for both A and B area were detected by EDS, and the results are listed in Table 3. 

From the results, it can be seen that the interface between the two nano-sized granules marked A and B is clear and the SAEDPs show that granule A is complete NiCr_2_O_4_ crystal and granule B is an amorphous structure of CMAS. The element composition dictated by EDS, as shown in Table 3, also proves that result, because the granule A is composed of 99% Ni, Cr, and O, and granule B is composed of 99% Ca, Mg, Al, Si, and O. This indicates that the chemical stability between NiCr_2_O_4_ and CMAS is relative high for such a strong contact (nano-size) and long interacting period (24 h).

The previous results show that NiCr_2_O_4_ ceramic has good chemical stability with molten CMAS, so they do not react under 1200 °C for a relatively long time (24 h). Chemical stability and non-wetting ability are the dominant factors that enable NiCr_2_O_4_ to work as an anti-CMAS material. The advantage of this kind of material is that it does not permit the formation of a reacting zone, thus arresting CMAS penetration and reducing the stress in the coating system. Furthermore, the thermal barrier ability of the coating can be maintained after CMAS attack, as it maintains a porous structure. 

## 4. Conclusions

The results show that three different types of NiCr_2_O_4_ material reveal many details on this issue. The reaction of nano-sized CMAS-NiCr_2_O_4_ mixed powder shows that NiCr_2_O_4_ has outstanding chemical stability with molten CMAS under 1200 °C, as predicted by the optical basicity analysis. No reaction was detected for a relatively long time (24 h). The morphology difference between the coating and bulk sample does not affect the chemical stability between CMAS and NiCr_2_O_4_. Also, the impurity of Cr_2_O_3_ (about 18.4%, according to intensity of peaks) was detected in the coating sample, indicating that a small amount of Cr_2_O_3_ in NiCr_2_O_4_ does not influence the chemical stability of NiCr_2_O_4_ with CMAS. Finally, both of the CMAS-contacting experiments the for coating and bulk sample show that NiCr_2_O_4_ and molten CMAS are non-wetting at 1200 °C, and there is not any element transportation across the border between them. The results indicate that NiCr_2_O_4_ can be used as a potential impermeable anti-CMAS material for the protection of TBCs. The methods of optical basicity and the analysis of CMAS-TBC reacting products could also be used in the search for other impermeable materials. 

## Figures and Tables

**Table 1:** Parameters of APS processing for NiCr_2_O_4_ deposition.

Primary Air (Ar, std. L/min)	Secondary Air (He, std. L/min)	Carrier Air (Ar, std. L/min)	Current (A)	Powder Rate (g/min)	Spray Distance (mm)
100	25	10	850	2	75

**Table 2:** EDS cation compositions (at %) of upper square (residue CMAS) and lower square (edge of NiCr_2_O_4_ coating) in Figure 2B.

At %	Ca	Mg	Al	Si	Ni	Cr	O
upper	16.05	1.86	5.84	21.73	-	-	51.56
lower	-	-	1.73	1.46	11.59	32.29	39.54

**Table 3:** EDS cation compositions (at %) of NiCr_2_O_4_ (area A) and CMAS (area B) in Figure 5A.

At %	Ca	Mg	Al	Si	Ni	Cr	O
A	0.72	0.05	0.04	0.93	11.44	29.99	56.79
B	11.85	10.89	2.32	22.83	0.46	0.23	51.39
